# Supplementary material for: Targeting Heparan Sulfate Proteoglycans as a Novel Therapeutic Strategy for Mucopolysaccharidoses
Source: Mol Ther Methods Clin Dev. 2018 Jun 18;10:8–16. doi: 10.1016/j.omtm.2018.05.002 (PMC6011039; doi:10.1016/j.omtm.2018.05.002)

OMTM, Volume *10*

Supplemental Information

Targeting Heparan Sulfate Proteoglycans as a Novel Therapeutic Strategy for Mucopolysaccharidoses

Valeria De Pasquale, Patrizia Sarogni, Valeria Pistorio, Giuliana Cerulo, Simona Paladino, and Luigi Michele Pavone

Supplementary Information

Title: Targeting heparan sulfate proteoglycans as a novel therapeutic strategy for mucopolysaccharidoses

Authors: Valeria De Pasquale, Patrizia Sarogni, Valeria Pistorio, Giuliana Cerulo, Simona Paladino, and Luigi Michele Pavone

Affiliations: Department of Molecular Medicine and Medical Biotechnology, University of Naples Federico II, 80131 Naples, Italy

Materials and Methods

Antibodies and reagents

Mouse anti-LAMP1 monoclonal antibody (555798) was purchased from BD Biosciences; mouse anti-β-actin monoclonal antibody (G043) from Abm; mouse anti-γ-tubulin antibody (T6557) from Sigma Aldrich; goat anti-mouse IgG polyclonal antibody conjugated to horseradish peroxidase (HRP) (sc-2031) from Santa Cruz Biotechnology; bovine serum albumin (BSA) (A7906), from Sigma Aldrich; SDS-PAGE reagents from Bio-Rad; fetal bovine serum (FBS) from GIBCO; Fibronectin (F2006) was purchased from Sigma Aldrich.

Cell cultures

Fibroblasts from MPS affected patients were kindly provided by the "Cell Line and DNA Biobank From Patients Affected by Genetic Diseases" (Istituto G. Gaslini, Genoa, Italy). Fibroblasts were cultured in Dulbecco's modified Eagle's medium (DMEM), supplemented with 10 % FBS, 2 mM L-glutamine, 100 Units/ml penicillin, and 100 μg/ml streptomycin, at 37 °C in a humidified 5% CO_2 atmosphere.

Measurement of radioactive glucosamine content into GAG chains

MPS fibroblasts were grown in normal medium supplemented with 7 μCi/ml ^{3}H-glucosamine (PerkinElmer) up to 80% confluence. The radioactive medium was removed and cells were incubated for 48 h in normal medium containing 2% FBS and 10^{-6} M of fibronectin. Cells were washed with PBS before harvesting, suspended in water, and lysed using freeze thaw cycles. An aliquot of cell lysate was taken out for the determination of protein concentration using the Lowry method. Lipids were extracted by addition of chloroform and methanol (chloroform-methanol-water 4:8:3, v/v/v). After 10 min of incubation at room temperature, extracts were recovered by centrifugation (10000 g for 10 min), washed with acetone, dried, and subjected to proteolysis overnight at 65 °C with 1 mg/ml papain in 100 mM sodium acetate buffer containing 5 mM EDTA and 5 mM cysteine (pH 5.5). The content of ^{3}H-glucosamine was measured by liquid scintillation counting, and normalized against protein concentration.

Western blotting

Protein extraction from cell lysates and immunoblot was performed as previously described. Briefly, fibroblasts were harvested in lysis buffer (50 mM Tris pH 7.5, 150 mM NaCl, 1 mM EDTA, 1 mM EGTA, 10 % glycerol, 1 % Triton-X-100, 1 mM β-glycerophosphate, 1 mM phenylmethylsulfonyl fluoride, protease inhibitor cocktail tablet, 1 mM sodium orthovanadate, 2.5 mM sodium pyrophosphate), incubated for 30 min on ice, and supernatants were collected and centrifuged for 10 min at 14,000 g. Protein concentration was estimated by Bradford assay, and 50

μg/lane of total proteins were separated on SDS gels and transferred to nitrocellulose membranes. Membranes were treated with a blocking buffer (25 mM Tris, pH 7.4, 200 mM NaCl, 0.5% Triton X-100) containing 5% non-fat powdered milk for 1 h at room temperature). Incubation with the primary antibody was carried out overnight at 4 °C. After serial washings, membranes were incubated with the HRP-conjugated secondary antibody for 1 h at room temperature. Following further washings of the membranes, chemiluminescence was generated by ECL system.

Statistical analysis

Data reported are expressed as the mean ± standard deviation (S.D.) of at least three separate experiments. Statistical significance was determined by Student's t-test. The value of $P < 0.05$ was considered to be statistically significant.

SUPPLEMENTARY FIGURE LEGENDS

Figure S1. A. Effect of fibronectin on ^{3}H-glucosamine content into GAGs of fibroblasts from MPS IIIB patients. Data reported are the means ± S.D. of three independent experiments performed in triplicate. *P<0.05. **B.** LAMP1 protein expression levels in untreated and NK1-treated MPS I and MPS IIIB fibroblasts as measured by Western blotting analysis. The upper blots were stripped and re-probed with anti-γ-tubulin or anti-β-actin antibodies to ensure equal loading of proteins in all lanes. The blots reported are representative of three independent experiments of equal design.

Figure S1

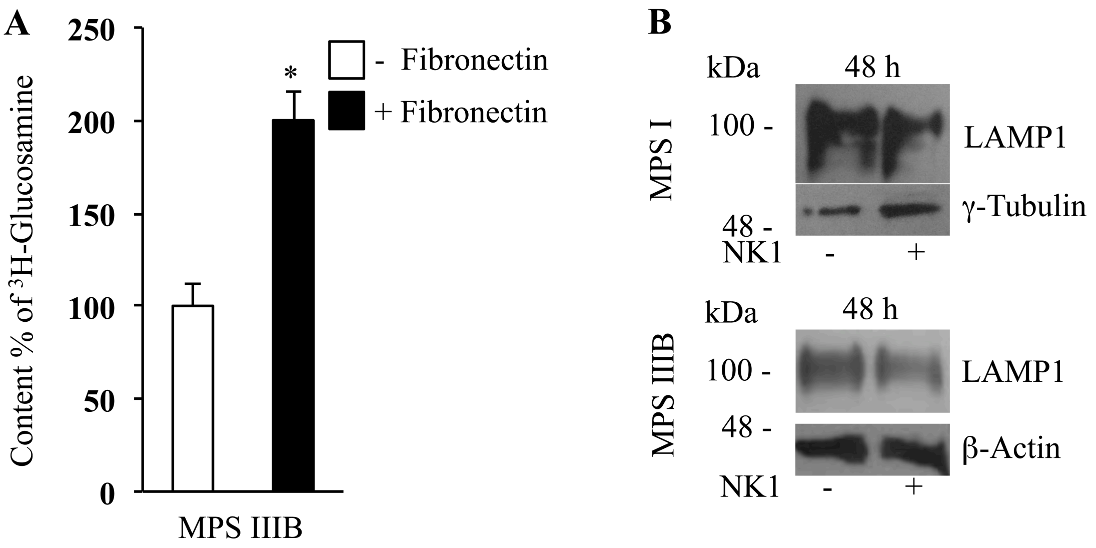

Supplement: Document S1. Supplemental Materials and Methods and Figure S1 [file mmc1.pdf]
