# Supplementary material for: Targeting Heparan Sulfate Proteoglycans as a Novel Therapeutic Strategy for Mucopolysaccharidoses
Source: Mol Ther Methods Clin Dev. 2018 Jun 18;10:8–16. doi: 10.1016/j.omtm.2018.05.002 (PMC6011039; doi:10.1016/j.omtm.2018.05.002)

Molecular Therapy
Methods & Clinical Development
Original Article

Targeting Heparan Sulfate Proteoglycans as a Novel Therapeutic Strategy for Mucopolysaccharidoses

Valeria De Pasquale,[1] Patrizia Sarogni,[1] Valeria Pistorio,[1] Giuliana Cerulo,[1] Simona Paladino,[1] and Luigi Michele Pavone[1]

[1]Department of Molecular Medicine and Medical Biotechnology, School of Medicine, University of Naples Federico II, 80131 Naples, Italy

Mucopolysaccharidoses (MPSs) are inherited metabolic diseases caused by the deficiency of lysosomal enzymes needed to catabolize glycosaminoglycans (GAGs). Four therapeutic options are currently considered: enzyme replacement therapy, substrate reduction therapy, gene therapy, and hematopoietic stem cell transplantation. However, while some of them exhibit limited clinical efficacy and require high costs, others are still in development. Therefore, alternative treatments for MPSs need to be explored. Here we describe an innovative therapeutic approach based on the use of a recombinant protein that is able to bind the excess of extracellular accumulated heparan sulfate (HS). We demonstrate that this protein is able to reduce lysosomal defects in primary fibroblasts from MPS I and MPS IIIB patients. We also show that, by masking the excess of extracellular accumulated HS in MPS fibroblasts, fibroblast growth factor (FGF) signal transduction can be positively modulated. We, therefore, suggest the use of a competitive binding molecule for HS in MPSs as an alternative strategy to prevent the detrimental extracellular substrate storage.

INTRODUCTION

Mucopolysaccharidoses (MPSs) are lysosomal storage diseases (LSDs) caused by mutations in genes encoding for lysosomal enzymes involved in the degradation of glycosaminoglycans (GAGs).[1] The accumulation of undigested GAGs results in the loss of cellular functions, tissue damage, and organ dysfunctions accounting for MPS clinical manifestations that include brain abnormalities and mental retardation; skeletal, joint, airway, and cardiac defects; and hearing and vision impairment. Affected patients usually die in their second or third decade of life. Depending on the accumulated GAGs, MPSs are classified into seven types (I, II, III, IV, VI, VII, and IX) that are variable in their prevalence, clinical symptoms, and degree of severity.[2]

Currently, therapeutic options for MPSs include enzyme replacement therapy (ERT), substrate reduction therapy (SRT), pharmacological chaperone therapy, gene therapy, and hematopoietic stem cells transplantation (HSCT).[3–6] Most of these treatments, showing variable and limited efficacy, are not curative, but they only ameliorate the symptoms of the disease. Thus, despite the recent undeniable advances in treatment outcomes for MPS diseases, many challenges still remain. Indeed, ERT, which is based on the administration of a recombinant enzyme replacing the deficient lysosomal one, is unable to correct the MPS-related neurological defects, due to the inability of recombinant enzymes to cross the blood-brain barrier.[7] Host immune responses as well as the failure to prevent neurological deterioration limit the utility of HSCT therapy for MPSs.[8,9] Despite improvements, the use of viral vectors in gene therapy is still in development, and it is in clinical trial for some MPS subtypes.[6,10–14] Due to the limits of these therapeutic strategies, research in progress is still focused on a better understanding of MPS physiopathology and development of more advanced therapeutic approaches.

Glycosaminoglycans are linear, negatively charged polysaccharides with molecular weights of about 10–100 kDa. There are two main types of GAGs: non-sulfated, which include hyaluronic acid (HA), and sulfated, which include chondroitin sulfate (CS), dermatan sulfate (DS), keratan sulfate (KS), heparin, and heparan sulfate (HS).[15] With the exception of HA, all GAGs are covalently attached to a core protein, forming the so-called proteoglycans that are abundantly found at the cell surface and in the extracellular matrix.[16] In particular, HS proteoglycans (HSPGs), either associated with the plasma membrane or localized in the extracellular matrix, modulate the activity of growth factors (GFs), such as fibroblast GF (FGF), vascular endothelial GF (VEGF), hepatocyte GF (HGF), and platelet-derived GF (PDGF), allowing their presentation to the cognate receptors in a biologically favorable form.[17–20] Another fundamental role of HSPGs is their contribution to the generation and long-range maintenance of morphogen gradients during embryogenesis, postnatal development, and regenerative processes.[21–25] Furthermore, HSPGs' interaction with adhesion molecules, receptor tyrosine kinases (RTKs), and Toll-like receptors accounts for their crucial role in

Received 22 February 2018; accepted 13 May 2018;
https://doi.org/10.1016/j.omtm.2018.05.002.

Correspondence: Luigi Michele Pavone, Department of Molecular Medicine and Medical Biotechnology, School of Medicine, University of Naples Federico II, Via S. Pansini 5, 80131 Naples, Italy.
E-mail: luigimichele.pavone@unina.it

 © 2018 The Author(s).
This is an open access article under the CC BY license (http://creativecommons.org/licenses/by/4.0/).

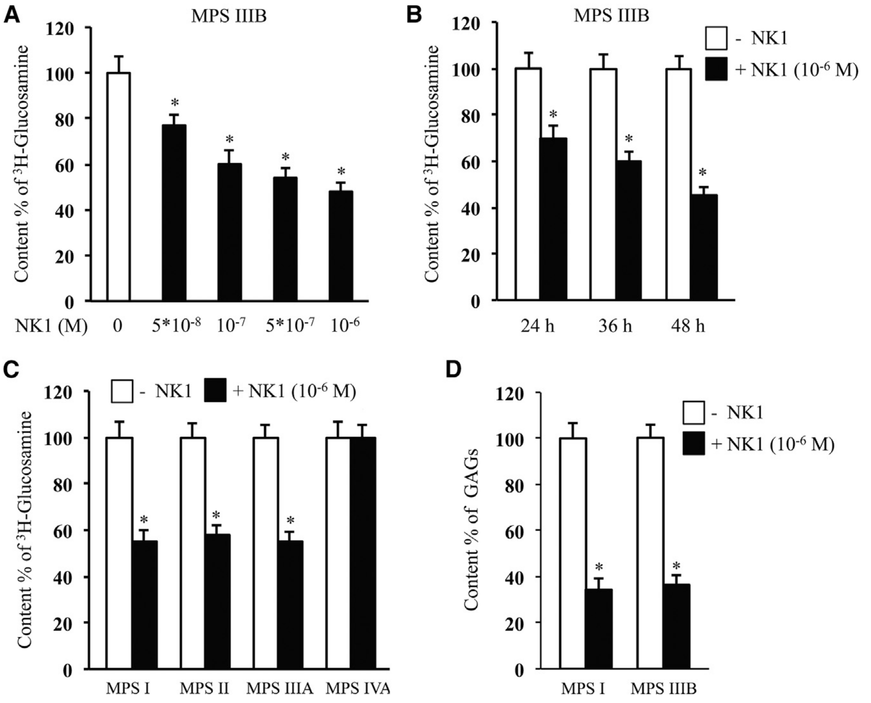

Figure 1. NK1 Reduces GAG Content in Fibroblasts from MPS-Affected Patients

(A and B) Dose- (A) and time- (B) dependent effect of NK1 on ^{3}H-glucosamine content into GAGs of fibroblasts from MPS IIIB-affected patients. Data reported are the means ± SD of three independent experiments performed in triplicate. *p < 0.05. (C) Effect of NK1 on ^{3}H-glucosamine content into GAGs of fibroblasts from patients affected by different MPS subtypes. Data reported are the means ± SD of three independent experiments performed in triplicate. *p < 0.05. (D) Effect of NK1 on GAG content in fibroblasts from MPS I and MPS IIIB patients assessed by Alcian blue method. Data reported are the means ± SD of three independent experiments performed in triplicate. *p < 0.05.

regulating cell adhesion and migration, proliferation, innate immunity, angiogenesis, apoptosis, and autophagy.[15–20,26]

Based on the functional relation between HSPGs and GFs, we developed an innovative approach for the treatment of MPS diseases, hereafter called substrate-masking technology, which uses a specific molecule with high binding affinity for the accumulated substrates (i.e., HS and/or DS). This technique enables us to restore the physiological equilibrium between morphogens or GFs, receptors, and HSPGs, allowing their proper interactions on the cell membrane and, in turn, activating downstream signaling. In particular, in this study we explored the potential therapeutic application of the hepatocyte GF/scatter factor (HGF/SF) natural spliced variant NK1,[27] which binds HS and DS with the same high affinity.[28] We evaluated the capability of NK1 to reduce HS content and consequent lysosomal abnormalities in fibroblasts from MPS I and MPS IIIB patients. Furthermore, we analyzed whether NK1 is able to modulate FGF-signaling activity in MPS I and IIIB primary fibroblasts. Our results provide the basis for the development of a potential strategy to restore signaling pathways disrupted in MPS diseases.

RESULTS AND DISCUSSION

NK1 Treatment Reduces GAG Content in Fibroblasts from MPS Patients

The autosomal recessive disorder MPS IIIB, caused by mutations in the gene encoding for the α-N-acetylglucosaminidase (NAGLU) enzyme, is one of the four MPS III (Sanfilippo syndrome) subtypes that are caused by the deficiency of lysosomal enzymes exclusively involved in the degradation of HS.[1] Current treatments for MPS III patients are limited to the clinical management of neurological symptoms.[29,30]

The first step for testing the efficacy of the substrate-masking technology was to investigate the ability of NK1 to reduce the quantity of accumulated GAGs *in vitro*, by evaluating ^{3}H-glucosamine content into MPS IIIB primary fibroblasts. GAG chains are composed of disaccharide-repeating units containing a uronic acid (D-glucuronic acid or L-iduronic acid) or a galactose and an amino sugar (D-galactosamine or D-glucosamine). While CS and DS contain galactosamine, heparin and HS contain glucosamine (GlcN), which is incorporated into GAGs after its conversion into glucosamine-6-phosphate (GlcN-6-P), N-acetyl-glucosamine-6-P (GlcNAc-6-P), GlcNAc-1-P, and UDP-alpha-GlcNAc.[26] Since HS is the only GAG accumulated in the MPS IIIB, ^{3}H-glucosamine levels represent a measure of the content of this specific GAG in primary fibroblasts from affected patients. MPS IIIB fibroblasts, grown to 80% confluence in the presence of ^{3}H-glucosamine, were incubated with increasing concentrations of NK1 for 48 hr, and the incorporated radionuclides were measured. A statistically significant reduction of ^{3}H-glucosamine content was observed at all NK1 tested doses as compared to untreated MPS IIIB fibroblasts. The effect of NK1 resulted in being dose dependent; however, at the highest concentration of NK1 (10^{-6} M), the reduction of ^{3}H-glucosamine content was up to 50% compared to untreated MPS IIIB fibroblasts (Figure 1A). At this concentration, NK1 neither interfered with cell growth and viability nor caused gross changes in cell morphology. Therefore, this dose was chosen for the time course study, in which ^{3}H-glucosamine content was measured over a time interval ranging from 24 to 48 hr. In this set of experiments, the effect of NK1 in reducing ^{3}H-glucosamine content into GAGs was already detectable after 24 hr, and it increased over time (Figure 1B). The major effects were detected at the dose of 10^{-6} M of NK1 after 48-hr treatment of the MPS IIIB fibroblasts; thus, we selected this dose and incubation time for further experiments.

Since NK1 binds with the same affinity both HS and DS,[28] we also evaluated, by the ^{3}H-glucosamine assay, the ability of NK1 to reduce GAG content in primary fibroblasts from MPS I, II, IIIA, and IVA patients. The results obtained showed a significant reduction of ^{3}H-glucosamine content in NK1-treated fibroblasts from patients affected by MPS I, II, and IIIA (Figure 1C), where the accumulated products are, respectively, HS and DS for MPS I and II and HS for MPS IIIA. Conversely, NK1 treatment of fibroblasts from patients affected by MPS IVA, in which the accumulated substrates are KS and CS, did not show any effect (Figure 1C), consistent with the fact that NK1 does not bind KS and CS.

Furthermore, the effect of NK1 on GAG storage levels after 48 hr of treatment was also measured by the Alcian blue method. This is a quantitative dye-binding assay commonly used for the *in vitro* analysis of sulfated GAGs,[31,32] based on the specific interaction between sulfated GAG polymers and the tetravalent cationic dye Alcian blue. The assay is performed at a low pH in order to neutralize all the carboxylic and phosphoric acid groups and at a high ionic strength to eliminate ionic interactions other than those between Alcian blue and sulfated GAGs. Indeed, hyaluronan, a non-sulfated GAG, does not react in this assay. As a result of our investigation, we found a significant decrease of GAG levels in MPS I and MPS IIIB fibroblasts treated with 10^{-6} M NK1 for 48 hr as compared to untreated fibroblasts (Figure 1D).

Moreover, in order to evaluate if HS-binding proteins[33,34] other than NK1 are able to reduce accumulated HS in MPS fibroblasts, we tested the capability of fibronectin to reduce GAG content in MPS IIIB fibroblasts. These cells, grown to 80% confluence in the presence of ^{3}H-glucosamine, were incubated for 48 hr with fibronectin at the same concentration of NK1, and the incorporated radionuclides were measured. The treatment with 10^{-6} M fibronectin resulted in a significant increase of ^{3}H-glucosamine content into GAGs in MPS IIIB fibroblasts as compared to untreated fibroblasts (Figure S1A).

These results demonstrate the efficacy and specificity of NK1 treatment in reducing GAG storage in the MPS subtypes characterized by an abnormal accumulation of HS and/or DS.

Rescue of the Lysosomal Defects in NK1-Treated Fibroblasts from MPS I and MPS IIIB Patients

In MPSs, the abnormal accumulation of undigested HS into lysosomes results in the enlargement of these organelles that start to occupy almost the whole cytoplasm.[35] To test whether NK1 was also able to reduce the lysosomal defects in MPS diseases, we incubated MPS I and MPS IIIB fibroblasts with 10^{-6} M NK1 for 48 hr, and we labeled lysosomes using LysoTraker. Quantitative confocal microscopy showed that the fluorescence intensity of the lysosomes was significantly reduced in NK1-treated fibroblasts compared to untreated ones (Figure 2A). We also labeled treated and untreated fibroblasts from MPS I and IIIB patients with the specific lysosomal-associated membrane protein 1 (LAMP1)[36] in order to evaluate the reduction of the lysosomal storage. Figure 2B shows prominent LAMP1 staining in untreated MPS I and MPS IIIB fibroblasts; upon treatment with 10^{-6} M NK1 for 48 hr, LAMP1 staining was significantly reduced. This observation was confirmed by western blotting analysis for LAMP1 in cell lysates from NK1-treated MPS I and MPS IIIB fibroblasts and untreated ones. Indeed, a reduction of LAMP1 protein levels was detected in both MPS I and MPS IIIB fibroblasts treated with NK1 as compared to untreated fibroblasts (Figure S1B).

Overall, these results demonstrate that cell treatment with NK1 results in a significant reduction of the lysosomal defects in MPS I and MPS IIIB fibroblasts.

Modulation of FGF Signaling by NK1 in MPS I and IIIB Fibroblasts

To verify whether NK1 was able to indirectly modulate FGF signaling by binding the extracellular HS, we performed a titration of the fibroblast GF receptor (FGFR) activation by stimulating starved MPS I and MPS IIIB fibroblasts with increasing doses of the human basic FGF (FGF2), both in the absence and in the presence of 10^{-6} M NK1. In particular, since activation of FGFR induces a variety of intracellular signaling cascades, including the MAPK/ERK pathway,[37,38] we evaluated by western blotting analysis the phosphorylation levels of ERK1/2 in untreated and NK1-treated fibroblasts from MPS I and III affected patients. The results obtained showed that FGF2 at the concentration of 10^{-12} M (Figure 3, lane 2, upper and lower blots) was unable to trigger ERK1/2 phosphorylation, whereas, in the presence of NK1, the same concentration of FGF2 promoted a significant phosphorylation of ERK1/2 (Figure 3, lane 5, upper and lower blots). These data suggest that the substrate-masking action of NK1 prevents the trapping of FGF2 by the excess of extracellular HS, thus increasing its availability and making FGF2 able to activate the FGFR-signaling cascade.

On the other hand, a reduction of ERK1/2 phosphorylation levels was observed at higher FGF2 concentrations (10^{-11} M and 10^{-10} M) in the presence of NK1 (Figure 3, lanes 6 and 7, upper and lower blots) as compared to un-pretreated cells (Figure 3, lanes 3 and 4, upper and lower blots). Indeed, in this case, the substrate-masking activity of NK1 limits the availability of HSPG-binding sites for FGF2 with a consequent decrease of ERK1/2 phosphorylation. Thus, even if FGF2 concentration would increase, in the presence of 10^{-6} M NK1, the effect on ERK1/2 phosphorylation will be always the same due to the same residual availability of HSPG. The administration of NK1 alone to the cells in the absence of FGF2 had no effect on ERK1/2 phosphorylation (Figure 3, lane 8, upper and lower blots), thus ruling out the potential ERK1/2 activation by NK1 itself.[39]

Overall, these data show that NK1 treatment in MPS fibroblasts is able to modulate FGF2-signaling activity by masking the excess of accumulated extracellular HS (Figure 4).

Conclusions

Our study describes an innovative potential strategy to rescue cell-signaling pathway alterations that contribute to MPS pathology.[40,41]

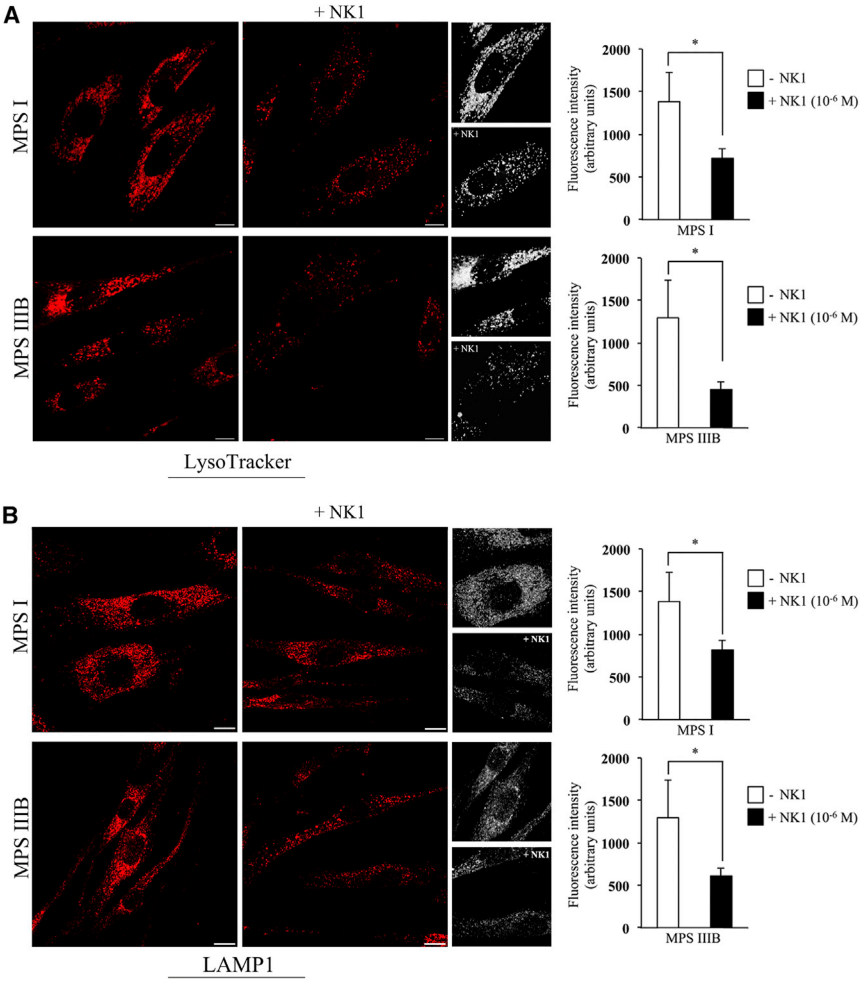

Figure 2. NK1 Reduces Lysosomal Aberration in Fibroblasts from MPS I and MPS IIIB Patients

(A) Representative images of lysosomes labeled with LysoTracker probe in untreated and NK1-treated fibroblasts from MPS I- and MPS IIIB-affected patients. 3D reconstructions of confocal sections along the z axis are shown (black and white panels). Scale bar, 10 μm. Quantitative analysis showed that, in NK1-treated fibroblasts, the fluorescence intensity of the lysosomes was significantly reduced as compared to the untreated fibroblasts, both in MPS I and MPS IIIB. Quantification of LysoTracker staining is the mean ± SD of three independent experiments. *$p < 0.05$. (B) Representative images of lysosomes labeled with antibody anti-LAMP1 in untreated and NK1-treated fibroblasts from MPS I- and MPS IIIB-affected patients. 3D reconstructions of confocal sections along the z axis are shown (black and white panels). Scale bar, 10 μm. Quantitative analysis showed that, in NK1-treated fibroblasts, the fluorescence intensity of the lysosomes was significantly reduced as compared to the untreated fibroblasts, both in MPS I and MPS IIIB. Quantification of LAMP1 fluorescence intensity is the mean ± SD of three independent experiments. *$p < 0.05$.

This strategy is based on the high binding affinity of the HGF/SF spliced variant NK1 for HS,[28] whose accumulation in cells and tissues is the main cause of MPS disease. The mesenchymal factor HGF/SF provides growth, motility, and morphogenic stimuli to epithelial, endothelial, and neural cells via exclusive activation of the tyrosine kinase receptor MET.[42] Although the affinity of HGF/SF for MET is very high,[43] subsequent activation of the receptor leading to sustained and effective downstream signaling is highly dependent upon GAG co-factors.[44–48] HS, heparin, and DS, but not CS, interact with HGF/SF and function as co-factors for HGF/SF.[49–53] The major HS and heparin-binding site of HGF/SF resides in its N-terminal domain.[28] Indeed, a heparin tetradecasaccharide has been co-crystallized with the truncated NK1 splice variant of HGF/SF, which only comprises the N-terminal domain, N, and the first Kringle domain, K1. The crystal structure identified the major GAG contacts as being in the N domain, and some additional contacts are made with the K1 domain.[54]

Here we demonstrate that NK1 is capable of reducing HS content in cultured fibroblasts from MPS-affected patients and, consequently, of reversing deregulated cellular processes, such as lysosomal defects. Furthermore, by this approach we have been able to support the previously recognized pathogenetic role of the extracellular accumulated substrate in MPS diseases. Indeed, it has been widely demonstrated that the accumulation of HS in MPS patients is not only restricted to the lysosome compartment but also the excess of HS is redistributed to different cellular (i.e., within the Golgi apparatus) and extracellular localizations.[55–59] In particular, a perturbation of the crucial HSPG-GF-receptor interactions may represent a general mechanism by which extracellular accumulated HS contributes to MPS pathogenesis.[60–62] Moreover, the excess of extracellular HS has been shown to impair FGF2 receptor binding and signaling in cells derived from MPS I patients[55] and to bind and sequester CXCL12-limiting hematopoietic migration in the murine model of MPS I.[56] Formation of the FGF2-FGFR-HSPG complex is necessary for mitogenesis and optimal biologic response to FGF2.[63–66] Accordingly, in this study we show that, in MPS-cultured fibroblasts, the excess of extracellular HSPGs sequesters FGF2 and inactivates its action. Indeed, our findings demonstrate that, by masking the excess of extracellular accumulated HSPGs, we are able to restore the physiological GF activity (Figure 4). Furthermore, in various injury and disease models, HGF and NK1 promote cell survival and tissue regeneration.[67] Although further *in vivo* studies to address the effectiveness of NK1 are needed, we envisage that this strategy may be of potential application to manage cell-signaling alterations occurring in MPS diseases.

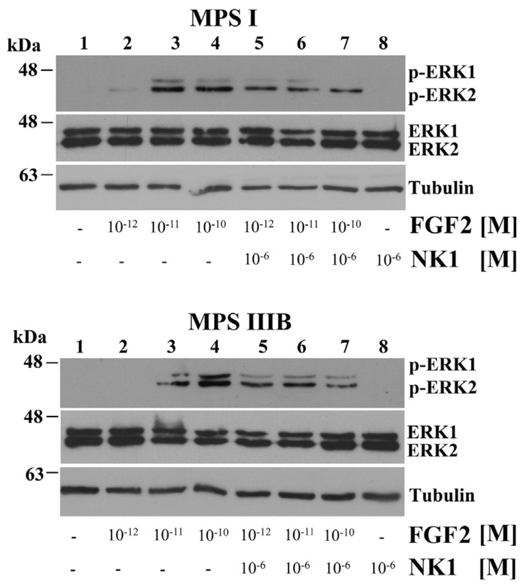

Figure 3. NK1 Treatment Restores FGF2 Activity in Fibroblasts from MPS I- and MPS IIIB-Affected Patients

Titration of FGF receptor activation was performed by stimulating for 10 min starved MPS I and MPS IIIB fibroblasts with increasing doses of FGF2, both in the absence and in the presence of 10^{-6} M NK1, and evaluating the phosphorylation levels of ERK1/2 by western blotting. The upper blots were stripped and re-probed with anti-ERK1/2 antibody. Anti-γ-tubulin antibody was used to ensure equal loading of proteins in all lanes. The blots reported are representative of three independent experiments of equal design.

MATERIALS AND METHODS

Antibodies and Reagents

Mouse anti-LAMP1 monoclonal antibody (555798) was purchased from BD Biosciences; mouse anti-diphosphorylated ERK1/2 monoclonal antibody (M8159) was from Sigma-Aldrich; rabbit anti-ERK1/2 polyclonal antibody (V114A) was from Promega; mouse anti-β-actin monoclonal antibody (G043) was from Abm; mouse anti-γ-tubulin antibody (T6557) was from Sigma-Aldrich; goat anti-mouse IgG polyclonal antibody conjugated to horseradish peroxidase (HRP) (sc-2031) and goat anti-rabbit IgG-HRP polyclonal antibody (sc-3837) were from Santa Cruz Biotechnology; goat anti-mouse IgG-TRITC antibody (T5393) was from Sigma-Aldrich; BSA (A7906) was from Sigma-Aldrich; SDS-PAGE reagents were from Bio-Rad; fetal bovine serum (FBS) was from Gibco; LysoTracker (L7528) was from Thermo Fisher Scientific; fibronectin (F2006) was from Sigma-Aldrich; FGF2 was from PeproTech; and Alcian blue dye (74240) was from EuroDiagnostica. The recombinant NK1 fragment of HGF was produced using yeast *Pichia pastoris* expression system and purified with heparin affinity chromatography as previously described.[39]

Cell Cultures

Fibroblasts from MPS-affected patients were kindly provided by the Cell Line and DNA Biobank from Patients Affected by Genetic Diseases (Istituto G. Gaslini, Genoa, Italy).[68] Fibroblasts were cultured in DMEM, supplemented with 10% FBS, 2 mM L-glutamine, 100 U/mL penicillin, and 100 μg/mL streptomycin, at 37°C in a humidified 5% CO_2 atmosphere.

Measurement of Radioactive Glucosamine Content into GAG Chains

MPS fibroblasts were grown in normal medium supplemented with 7 μCi/mL ^{3}H-glucosamine (PerkinElmer) up to 80% confluence. The radioactive medium was removed, and cells were incubated for 24, 36, and 48 hr in normal medium containing 2% FBS and 0, 5×10^{-8}, 10^{-7}, 5×10^{-7}, 10^{-6} M NK1. Cells were washed with PBS before harvesting, suspended in water, and lysed using freeze-thaw cycles. An aliquot of cell lysate was taken out for the determination of protein concentration using the Lowry method.[69] Lipids were extracted by the addition of chloroform and methanol (chloroform-methanol-water 4:8:3, v/v/v). After 10-min of incubation at room temperature, extracts were recovered by centrifugation (10,000 × *g* for 10 min), washed with acetone, dried, and subjected to proteolysis overnight at 65°C with 1 mg/mL papain in 100 mM sodium acetate buffer containing 5 mM EDTA and 5 mM cysteine (pH 5.5). The content of ^{3}H-glucosamine was measured by liquid scintillation counting and normalized against protein concentration.

Assessment of GAG Levels

Accumulation of GAGs was estimated with Alcian blue reagent using sulfated GAG quantitative kit Wieslab.[31] This method is used to detect sulfated GAG in biological samples, tissue, and cells extracts. The Alcian blue reagent in Wieslab's sulfated GAG assay has been carefully selected and optimized for this particular use. There is no interference from proteins or nucleic acids in this method, in contrast to the dimethylmethylene blue (DMMB) method or other dye-binding methods.[32] In brief, cells were plated in a number of 1.5×10^5 per well in 6-well plates and incubated overnight to allow the attachment. Next, cells were supplemented with normal medium containing 2% FBS and 10^{-6} M NK1 or PBS. After 48 hr of incubation, harvested cells were digested overnight at 65°C with 1 mg/mL papain in 100 mM sodium acetate buffer containing 5 mM EDTA and 5 mM cysteine (pH 5.5). GAG content and protein concentration were estimated, respectively, with Alcian blue and Lowry method according to the manufacturer's protocols.[32,69] GAG content was expressed per protein amount (μg/mg of protein) and normalized with respect to untreated fibroblasts.

Fluorescence Microscopy

LysoTracker was used to label lysosomes.[70] Briefly, untreated and treated fibroblasts with 10^{-6} M NK1 for 48 hr, grown on a coverslip, were incubated with LysoTracker probe for 1 hr at 37°C, then washed with PBS, and fixed with 4% paraformaldehyde (PFA) solution in PBS. After washing with PBS, the coverslips were mounted with 1:1 PBS:glycerol solution and then observed under a confocal fluorescence microscope.

For LAMP1 fluorescent staining, untreated and treated fibroblasts with 10^{-6} M NK1 for 48 hr, grown on a coverslip, were washed with PBS, fixed with 4% PFA, and quenched with 50 mM NH_4Cl.

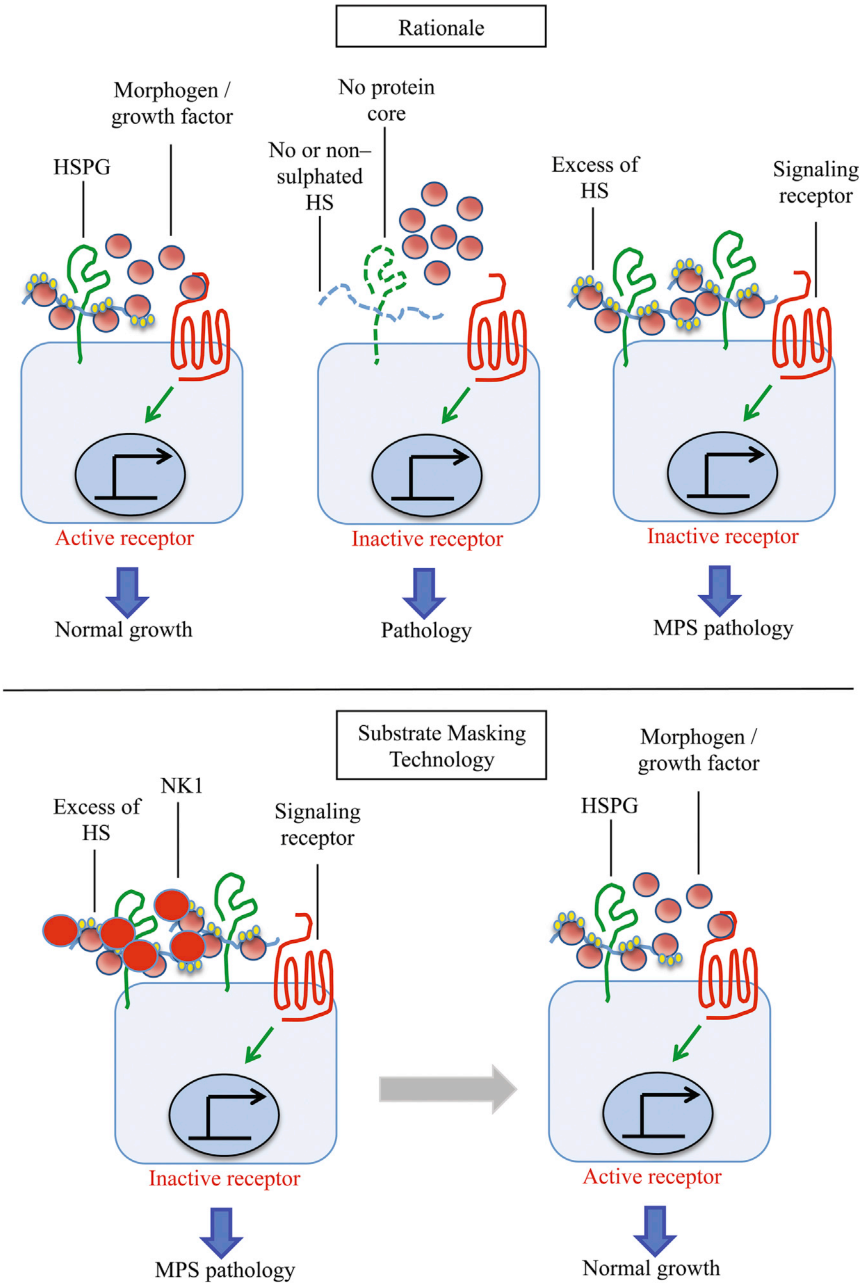

Figure 4. Schematic Diagram Depicting the Rationale and the Molecular Mechanism of the Substrate-Masking Technology for the Treatment of MPS Diseases

(Modified from Häcker et al.[75]) The rationale for the substrate-masking technology is based on the evidence that the equilibrium between HSPGs, morphogens or growth factors, and receptors accounts for the physiological receptor-signaling activation. On the contrary, when HSPG levels on cell membrane are accumulated, signaling receptor activation is diminished due to the increased binding activity of the morphogens or growth factors to the additional GAG chains, thus leading to MPS pathology. A recombinant protein with a high binding affinity for HS and DS saturating the excess of extracellular GAGs may restore the physiological equilibrium between HSPGs, morphogens or growth factors, and receptors and the subsequent signaling.

Then, cells were permeabilized with 0.2% Triton X-100 for 5 min and blocked for 30 min in PBS containing 10% FBS and 1% BSA. Fibroblasts were then incubated for 1 hr with anti-LAMP1 primary antibody that was detected with an anti-mouse IgG-TRITC secondary antibody. After washing with PBS, the coverslips were mounted with 1:1 PBS:glycerol solution and then observed under a confocal fluorescence microscope.

Images were collected using a laser-scanning confocal microscope (LSM 510; Carl Zeiss Microimaging) equipped with a planapo 63× oil immersion (numerical aperture [NA] 1.4) objective lens by using the appropriate laser lines. Images were acquired with the confocal pinhole set to one Airy unit, taking Z-slices from the top to the bottom of the cell by using the same setting (laser power, detector gain), as well as the same threshold of fluorescence intensity in all experimental conditions (untreated and NK1-treated fibroblasts). Quantification analyses were carried out using LSM 510 software. In particular, the mean fluorescence intensities were measured by drawing regions of interest (ROIs) around the entire cell and corrected for background in random chosen area.

Western Blotting

Protein extraction from cell lysates and immunoblot were performed as previously described.[71,72] Briefly, fibroblasts were harvested in lysis buffer (50 mM Tris [pH 7.5], 150 mM NaCl, 1 mM EDTA, 1 mM EGTA, 10% glycerol, 1% Triton X-100, 1 mM β-glycerophosphate, 1 mM phenylmethylsulfonyl fluoride, protease inhibitor cocktail tablet, 1 mM sodium orthovanadate, and 2.5 mM sodium pyrophosphate), incubated for 30 min on ice, and supernatants were collected and centrifuged for 10 min at 14,000 × *g*. Protein concentration was estimated by Bradford assay, and 50 μg/lane total proteins was separated on SDS gels and transferred to nitrocellulose membranes.[73] Membranes were treated with a blocking buffer (25 mM Tris [pH 7.4], 200 mM NaCl, and 0.5% Triton X-100) containing 5% non-fat powdered milk for 1 hr at room temperature.[74] Incubation with the primary antibody was carried out overnight at 4°C. After serial washings, membranes were incubated with the HRP-conjugated secondary antibody for 1 hr at room temperature. Following

further washings of the membranes, chemiluminescence was generated by enhanced chemiluminescence (ECL) system.

Statistical Analysis

Data reported are expressed as the mean ± SD of at least three separate experiments. Statistical significance was determined by Student's t test. The value of $p < 0.05$ was considered to be statistically significant.

SUPPLEMENTAL INFORMATION

Supplemental Information includes Supplemental Materials and Methods and one figure and can be found with this article online at https://doi.org/10.1016/j.omtm.2018.05.002.

AUTHOR CONTRIBUTIONS

L.M.P. conceived the study. V.D.P., P.S., V.P., G.C., S.P., and L.M.P. designed and carried out the experiments and analyzed the data. L.M.P. wrote the manuscript with input from all the other authors. All authors approved the manuscript.

CONFLICTS OF INTEREST

L.M.P. has licensed compositions comprising hepatocyte growth factor or variants thereof for use in the treatment of mucopolysaccharidoses (granted Italian patent MI2014A001454). The authors declare no additional competing financial interests.

ACKNOWLEDGMENTS

This work was supported by funds from the University of Naples Federico II to L.M.P. The Cell Line and DNA Biobank from Patients Affected by Genetic Diseases (Istituto G. Gaslini), member of the Telethon Network of Genetic Biobanks (project GTB12001), funded by Telethon Italy, provided us with specimens (fibroblasts from MPS patients). We acknowledge helpful comments from Professor Enrico Moro (University of Padova, Italy).

REFERENCES

1. Neufeld, E.F., and Muenzer, J. (2001). The mucopolysaccharidoses. In The Metabolic and Molecular Bases of Inherited Disease, Eighth Editon, C.R. Scriver, A.L. Beaudet, W.S. Sly, and D. Valle, eds. (New York: McGraw-Hill), pp. 3421–3452.
2. Clarke, L.A. (2008). The mucopolysaccharidoses: a success of molecular medicine. Expert Rev. Mol. Med. *10*, e1.
3. Noh, H., and Lee, J.I. (2014). Current and potential therapeutic strategies for mucopolysaccharidoses. J. Clin. Pharm. Ther. *39*, 215–224.
4. Hollak, C.E., and Wijburg, F.A. (2014). Treatment of lysosomal storage disorders: successes and challenges. J. Inherit. Metab. Dis. *37*, 587–598.
5. Parenti, G., Andria, G., and Ballabio, A. (2015). Lysosomal storage diseases: from pathophysiology to therapy. Annu. Rev. Med. *66*, 471–486.
6. Poswar, F., Baldo, G., and Giugliani, R. (2017). Phase I and II clinical trials for the mucopolysaccharidoses. Expert Opin. Investig. Drugs *26*, 1331–1340.
7. Muenzer, J. (2014). Early initiation of enzyme replacement therapy for the mucopolysaccharidoses. Mol. Genet. Metab. *111*, 63–72.
8. Lutzko, C., Kruth, S., Abrams-Ogg, A.C., Lau, K., Li, L., Clark, B.R., Ruedy, C., Nanji, S., Foster, R., Kohn, D., et al. (1999). Genetically corrected autologous stem cells engraft, but host immune responses limit their utility in canine alpha-L-iduronidase deficiency. Blood *93*, 1895–1905.
9. Welling, L., Marchal, J.P., van Hasselt, P., van der Ploeg, A.T., Wijburg, F.A., and Boelens, J.J. (2015). Early umbilical cord blood-derived stem cell transplantation does not prevent neurological deterioration in mucopolysaccharidosis type III. JIMD Rep. *18*, 63–68.
10. Shull, R., Lu, X., Dubé, I., Lutzko, C., Kruth, S., Abrams-Ogg, A., Kiem, H.P., Goehle, S., Schuening, F., Millan, C., and Carter, R. (1996). Humoral immune response limits gene therapy in canine MPS I. Blood *88*, 377–379.
11. Ferla, R., Alliegro, M., Marteau, J.B., Dell'Anno, M., Nusco, E., Pouillot, S., Galimberti, S., Valsecchi, M.G., Zuliani, V., and Auricchio, A. (2017). Non-clinical safety and efficacy of an AAV2/8 vector administered intravenously for treatment of Mucopolysaccharidosis type VI. Mol. Ther. Methods Clin. Dev. *6*, 143–158.
12. Sawamoto, K., Chen, H.H., Alméciga-Díaz, C.J., Mason, R.W., and Tomatsu, S. (2018). Gene therapy for Mucopolysaccharidoses. Mol. Genet. Metab. *123*, 59–68.
13. Tardieu, M., Zérah, M., Gougeon, M.L., Ausseil, J., de Bournonville, S., Husson, B., Zafeiriou, D., Parenti, G., Bourget, P., Poirier, B., et al. (2017). Intracerebral gene therapy in children with mucopolysaccharidosis type IIIB syndrome: an uncontrolled phase 1/2 clinical trial. Lancet Neurol. *16*, 712–720.
14. Tardieu, M., Zérah, M., Husson, B., de Bournonville, S., Deiva, K., Adamsbaum, C., Vincent, F., Hocquemiller, M., Broissand, C., Furlan, V., et al. (2014). Intracerebral administration of adeno-associated viral vector serotype rh.10 carrying human SGSH and SUMF1 cDNAs in children with mucopolysaccharidosis type IIIA disease: results of a phase I/II trial. Hum. Gene Ther. *25*, 506–516.
15. Jackson, R.L., Busch, S.J., and Cardin, A.D. (1991). Glycosaminoglycans: molecular properties, protein interactions, and role in physiological processes. Physiol. Rev. *71*, 481–539.
16. Iozzo, R.V., and Schaefer, L. (2015). Proteoglycan form and function: A comprehensive nomenclature of proteoglycans. Matrix Biol. *42*, 11–55.
17. Matsuo, I., and Kimura-Yoshida, C. (2013). Extracellular modulation of Fibroblast Growth Factor signaling through heparan sulfate proteoglycans in mammalian development. Curr. Opin. Genet. Dev. *23*, 399–407.
18. Billings, P.C., and Pacifici, M. (2015). Interactions of signaling proteins, growth factors and other proteins with heparan sulfate: mechanisms and mysteries. Connect. Tissue Res. *56*, 272–280.
19. Kim, S.H., Turnbull, J., and Guimond, S. (2011). Extracellular matrix and cell signalling: the dynamic cooperation of integrin, proteoglycan and growth factor receptor. J. Endocrinol. *209*, 139–151.
20. Kresse, H., and Schönherr, E. (2001). Proteoglycans of the extracellular matrix and growth control. J. Cell. Physiol. *189*, 266–274.
21. Lin, X. (2004). Functions of heparan sulfate proteoglycans in cell signaling during development. Development *131*, 6009–6021.
22. Poulain, F.E., and Yost, H.J. (2015). Heparan sulfate proteoglycans: a sugar code for vertebrate development? Development *142*, 3456–3467.
23. Hufnagel, L., Kreuger, J., Cohen, S.M., and Shraiman, B.I. (2006). On the role of glypicans in the process of morphogen gradient formation. Dev. Biol. *300*, 512–522.
24. Chung, H., Multhaupt, H.A., Oh, E.S., and Couchman, J.R. (2016). Minireview: Syndecans and their crucial roles during tissue regeneration. FEBS Lett. *590*, 2408–2417.
25. Patel, V.N., Pineda, D.L., and Hoffman, M.P. (2017). The function of heparan sulfate during branching morphogenesis. Matrix Biol. *57-58*, 311–323.
26. Gandhi, N.S., and Mancera, R.L. (2008). The structure of glycosaminoglycans and their interactions with proteins. Chem. Biol. Drug Des. *72*, 455–482.
27. Cioce, V., Csaky, K.G., Chan, A.M., Bottaro, D.P., Taylor, W.G., Jensen, R., Aaronson, S.A., and Rubin, J.S. (1996). Hepatocyte growth factor (HGF)/NK1 is a naturally occurring HGF/scatter factor variant with partial agonist/antagonist activity. J. Biol. Chem. *271*, 13110–13115.
28. Deakin, J.A., Blaum, B.S., Gallagher, J.T., Uhrín, D., and Lyon, M. (2009). The binding properties of minimal oligosaccharides reveal a common heparan sulfate/dermatan sulfate-binding site in hepatocyte growth factor/scatter factor that can accommodate a wide variety of sulfation patterns. J. Biol. Chem. *284*, 6311–6321.
29. Fedele, A.O. (2015). Sanfilippo syndrome: causes, consequences, and treatments. Appl. Clin. Genet. *8*, 269–281.

30. Ghosh, A., Shapiro, E., Rust, S., Delaney, K., Parker, S., Shaywitz, A.J., Morte, A., Bubb, G., Cleary, M., Bo, T., et al. (2017). Recommendations on clinical trial design for treatment of Mucopolysaccharidosis Type III. Orphanet J. Rare Dis. *12*, 117.

31. Moskot, M., Jakóbkiewicz-Banecka, J., Kloska, A., Smolińska, E., Mozolewski, P., Malinowska, M., Rychłowski, M., Banecki, B., Węgrzyn, G., and Gabig-Cimińska, M. (2015). Modulation of expression of genes involved in glycosaminoglycan metabolism and lysosome biogenesis by flavonoids. Sci. Rep. *5*, 9378.

32. Björnsson, S. (1993). Simultaneous preparation and quantitation of proteoglycans by precipitation with alcian blue. Anal. Biochem. *210*, 282–291.

33. Dreyfuss, J.L., Regatieri, C.V., Jarrouge, T.R., Cavalheiro, R.P., Sampaio, L.O., and Nader, H.B. (2009). Heparan sulfate proteoglycans: structure, protein interactions and cell signaling. An. Acad. Bras. Cienc. *81*, 409–429.

34. Heremans, A., De Cock, B., Cassiman, J.J., Van den Berghe, H., and David, G. (1990). The core protein of the matrix-associated heparan sulfate proteoglycan binds to fibronectin. J. Biol. Chem. *265*, 8716–8724.

35. Mizumoto, S., Ikegawa, S., and Sugahara, K. (2013). Human genetic disorders caused by mutations in genes encoding biosynthetic enzymes for sulfated glycosaminoglycans. J. Biol. Chem. *288*, 10953–10961.

36. Carlsson, S.R., and Fukuda, M. (1990). The polylactosaminoglycans of human lysosomal membrane glycoproteins lamp-1 and lamp-2. Localization on the peptide backbones. J. Biol. Chem. *265*, 20488–20495.

37. Wang, Z., Wang, Y., Ye, J., Lu, X., Cheng, Y., Xiang, L., Chen, L., Feng, W., Shi, H., Yu, X., et al. (2015). bFGF attenuates endoplasmic reticulum stress and mitochondrial injury on myocardial ischaemia/reperfusion via activation of PI3K/Akt/ERK1/2 pathway. J. Cell. Mol. Med. *19*, 595–607.

38. Chua, C.C., Rahimi, N., Forsten-Williams, K., and Nugent, M.A. (2004). Heparan sulfate proteoglycans function as receptors for fibroblast growth factor-2 activation of extracellular signal-regulated kinases 1 and 2. Circ. Res. *94*, 316–323.

39. Pavone, L.M., Cattaneo, F., Rea, S., De Pasquale, V., Spina, A., Sauchelli, E., Mastellone, V., and Ammendola, R. (2011). Intracellular signaling cascades triggered by the NK1 fragment of hepatocyte growth factor in human prostate epithelial cell line PNT1A. Cell. Signal. *23*, 1961–1971.

40. Costa, R., Urbani, A., Salvalaio, M., Bellesso, S., Cieri, D., Zancan, I., Filocamo, M., Bonaldo, P., Szabò, I., Tomanin, R., and Moro, E. (2017). Perturbations in cell signaling elicit early cardiac defects in mucopolysaccharidosis type II. Hum. Mol. Genet. *26*, 1643–1655.

41. Kingma, S.D.K., Wagemans, T., IJlst, L., Bronckers, A.L.J.J., van Kuppevelt, T.H., Everts, V., Wijburg, F.A., and van Vlies, N. (2016). Altered interaction and distribution of glycosaminoglycans and growth factors in mucopolysaccharidosis type I bone disease. Bone *88*, 92–100.

42. Trusolino, L., Bertotti, A., and Comoglio, P.M. (2010). MET signalling: principles and functions in development, organ regeneration and cancer. Nat. Rev. Mol. Cell Biol. *11*, 834–848.

43. Mark, M.R., Lokker, N.A., Zioncheck, T.F., Luis, E.A., and Godowski, P.J. (1992). Expression and characterization of hepatocyte growth factor receptor-IgG fusion proteins. Effects of mutations in the potential proteolytic cleavage site on processing and ligand binding. J. Biol. Chem. *267*, 26166–26171.

44. Xu, D., and Esko, J.D. (2014). Demystifying heparan sulfate-protein interactions. Annu. Rev. Biochem. *83*, 129–157.

45. Deakin, J.A., and Lyon, M. (1999). Differential regulation of hepatocyte growth factor/scatter factor by cell surface proteoglycans and free glycosaminoglycan chains. J. Cell Sci. *112*, 1999–2009.

46. Lyon, M., Deakin, J.A., and Gallagher, J.T. (2002). The mode of action of heparan and dermatan sulfates in the regulation of hepatocyte growth factor/scatter factor. J. Biol. Chem. *277*, 1040–1046.

47. Rubin, J.S., Day, R.M., Breckenridge, D., Atabey, N., Taylor, W.G., Stahl, S.J., Wingfield, P.T., Kaufman, J.D., Schwall, R., and Bottaro, D.P. (2001). Dissociation of heparan sulfate and receptor binding domains of hepatocyte growth factor reveals that heparan sulfate-c-met interaction facilitates signaling. J. Biol. Chem. *276*, 32977–32983.

48. Sergeant, N., Lyon, M., Rudland, P.S., Fernig, D.G., and Delehedde, M. (2000). Stimulation of DNA synthesis and cell proliferation of human mammary myoepithelial-like cells by hepatocyte growth factor/scatter factor depends on heparan sulfate proteoglycans and sustained phosphorylation of mitogen-activated protein kinases p42/44. J. Biol. Chem. *275*, 17094–17099.

49. Lyon, M., Deakin, J.A., Mizuno, K., Nakamura, T., and Gallagher, J.T. (1994). Interaction of hepatocyte growth factor with heparan sulfate. Elucidation of the major heparan sulfate structural determinants. J. Biol. Chem. *269*, 11216–11223.

50. Rahmoune, H., Rudland, P.S., Gallagher, J.T., and Fernig, D.G. (1998). Hepatocyte growth factor/scatter factor has distinct classes of binding site in heparan sulfate from mammary cells. Biochemistry *37*, 6003–6008.

51. Lyon, M., Deakin, J.A., Rahmoune, H., Fernig, D.G., Nakamura, T., and Gallagher, J.T. (1998). Hepatocyte growth factor/scatter factor binds with high affinity to dermatan sulfate. J. Biol. Chem. *273*, 271–278.

52. Béchard, D., Gentina, T., Delehedde, M., Scherpereel, A., Lyon, M., Aumercier, M., Vazeux, R., Richet, C., Degand, P., Jude, B., et al. (2001). Endocan is a novel chondroitin sulfate/dermatan sulfate proteoglycan that promotes hepatocyte growth factor/scatter factor mitogenic activity. J. Biol. Chem. *276*, 48341–48349.

53. Sakata, H., Stahl, S.J., Taylor, W.G., Rosenberg, J.M., Sakaguchi, K., Wingfield, P.T., and Rubin, J.S. (1997). Heparin binding and oligomerization of hepatocyte growth factor/scatter factor isoforms. Heparan sulfate glycosaminoglycan requirement for Met binding and signaling. J. Biol. Chem. *272*, 9457–9463.

54. Ultsch, M., Lokker, N.A., Godowski, P.J., and de Vos, A.M. (1998). Crystal structure of the NK1 fragment of human hepatocyte growth factor at 2.0 A resolution. Structure *6*, 1383–1393.

55. Pan, C., Nelson, M.S., Reyes, M., Koodie, L., Brazil, J.J., Stephenson, E.J., Zhao, R.C., Peters, C., Selleck, S.B., Stringer, S.E., and Gupta, P. (2005). Functional abnormalities of heparan sulfate in mucopolysaccharidosis-I are associated with defective biologic activity of FGF-2 on human multipotent progenitor cells. Blood *106*, 1956–1964.

56. Watson, H.A., Holley, R.J., Langford-Smith, K.J., Wilkinson, F.L., van Kuppevelt, T.H., Wynn, R.F., Wraith, J.E., Merry, C.L., and Bigger, B.W. (2014). Heparan sulfate inhibits hematopoietic stem and progenitor cell migration and engraftment in mucopolysaccharidosis I. J. Biol. Chem. *289*, 36194–36203.

57. De Pasquale, V., Pezone, A., Sarogni, P., Tramontano, A., Schiattarella, G.G., Avvedimento, V.E., Paladino, S., and Pavone, L.M. (2018). EGFR activation triggers cellular hypertrophy and lysosomal disease in NAGLU-depleted cardiomyoblasts, mimicking the hallmarks of mucopolysaccharidosis IIIB. Cell Death Dis. *9*, 40.

58. Holley, R.J., Deligny, A., Wei, W., Watson, H.A., Niñonuevo, M.R., Dagälv, A., Leary, J.A., Bigger, B.W., Kjellén, L., and Merry, C.L. (2011). Mucopolysaccharidosis type I, unique structure of accumulated heparan sulfate and increased N-sulfotransferase activity in mice lacking α-l-iduronidase. J. Biol. Chem. *286*, 37515–37524.

59. Dwyer, C.A., Scudder, S.L., Lin, Y., Dozier, L.E., Phan, D., Allen, N.J., Patrick, G.N., and Esko, J.D. (2017). Neurodevelopmental changes in excitatory synaptic structure and function in the cerebralcortex of Sanfilippo syndrome IIIA mice. Sci. Rep. *7*, 46576.

60. Heppner, J.M., Zaucke, F., and Clarke, L.A. (2015). Extracellular matrix disruption is an early event in the pathogenesis of skeletal disease in mucopolysaccharidosis I. Mol. Genet. Metab. *114*, 146–155.

61. Bishop, J.R., Schuksz, M., and Esko, J.D. (2007). Heparan sulphate proteoglycans fine-tune mammalian physiology. Nature *446*, 1030–1037.

62. Vlodavsky, I., Miao, H.Q., Medalion, B., Danagher, P., and Ron, D. (1996). Involvement of heparan sulfate and related molecules in sequestration and growth promoting activity of fibroblast growth factor. Cancer Metastasis Rev. *15*, 177–186.

63. Guglier, S., Hricovíni, M., Raman, R., Polito, L., Torri, G., Casu, B., Sasisekharan, R., and Guerrini, M. (2008). Minimum FGF2 binding structural requirements of heparin and heparan sulfate oligosaccharides as determined by NMR spectroscopy. Biochemistry *47*, 13862–13869.

64. Klagsbrun, M. (1990). The affinity of fibroblast growth factors (FGFs) for heparin; FGF-heparan sulfate interactions in cells and extracellular matrix. Curr. Opin. Cell Biol. *2*, 857–863.

65. Oussoren, E., Brands, M.M., Ruijter, G.J., der Ploeg, A.T., and Reuser, A.J. (2011). Bone, joint and tooth development in mucopolysaccharidoses: relevance to therapeutic options. Biochim. Biophys. Acta *1812*, 1542–1556.

66. Bellesso, S., Salvalaio, M., Lualdi, S., Tognon, E., Costa, R., Braghetta, P., Giraudo, C., Stramare, R., Rigon, L., Filocamo, M., et al. (2018). FGF signaling deregulation is associated with early developmental skeletal defects in animal models for mucopolysaccharidosis type II (MPSII). Hum. Mol. Genet. , Published online April 10, 2018. https://doi.org/10.1093/hmg/ddy131.

67. Nakamura, T., Sakai, K., Nakamura, T., and Matsumoto, K. (2011). Hepatocyte growth factor twenty years on: Much more than a growth factor. J. Gastroenterol. Hepatol. *26* (*Suppl 1*), 188–202.

68. Filocamo, M., Mazzotti, R., Corsolini, F., Stroppiano, M., Stroppiana, G., Grossi, S., Lualdi, S., Tappino, B., Lanza, F., Galotto, S., and Biancheri, R. (2014). Cell line and DNA biobank from patients affected by genetic diseases. Open J. Bioresour. *1*, e2.

69. Waterborg, J.H. (2002). The Lowry method for protein quantitation. In The Protein Protocols Handbook, Second Edition, J.M. Walker, ed. (Totowa, NJ: Humana Press), pp. 7–9.

70. Chazotte, B. (2011). Labeling membranes with fluorescent phosphatidylethanolamine. Cold Spring Harb. Protoc. *2011*, pdb.prot5621.

71. Schiattarella, G.G., Cerulo, G., De Pasquale, V., Cocchiaro, P., Paciello, O., Avallone, L., Belfiore, M.P., Iacobellis, F., Di Napoli, D., Magliulo, F., et al. (2015). The murine model of Mucopolysaccharidosis IIIB develops cardiopathies over time leading to heart failure. PLoS ONE *10*, e0131662.

72. Auriemma, C., Viscardi, M., Tafuri, S., Pavone, L.M., Capuano, F., Rinaldi, L., Della Morte, R., Iovane, G., and Staiano, N. (2010). Integrin receptors play a role in the internalin B-dependent entry of Listeria monocytogenes into host cells. Cell. Mol. Biol. Lett. *15*, 496–506.

73. Pavone, L.M., Rea, S., Trapani, F., De Pasquale, V., Tafuri, S., Papparella, S., and Paciello, O. (2012). Role of serotonergic system in the pathogenesis of fibrosis in canine idiopathic inflammatory myopathies. Neuromuscul. Disord. *22*, 549–557.

74. Spina, A., Rea, S., De Pasquale, V., Mastellone, V., Avallone, L., and Pavone, L.M. (2011). Fate map of serotonin transporter-expressing cells in developing mouse thyroid. Anat. Rec. (Hoboken) *294*, 384–390.

75. Häcker, U., Nybakken, K., and Perrimon, N. (2005). Heparan sulphate proteoglycans: the sweet side of development. Nat. Rev. Mol. Cell Biol. *6*, 530–541.

OMTM, Volume *10*

Supplemental Information

Targeting Heparan Sulfate Proteoglycans as a Novel Therapeutic Strategy for Mucopolysaccharidoses

Valeria De Pasquale, Patrizia Sarogni, Valeria Pistorio, Giuliana Cerulo, Simona Paladino, and Luigi Michele Pavone

Supplementary Information

Title: Targeting heparan sulfate proteoglycans as a novel therapeutic strategy for mucopolysaccharidoses

Authors: Valeria De Pasquale, Patrizia Sarogni, Valeria Pistorio, Giuliana Cerulo, Simona Paladino, and Luigi Michele Pavone

Affiliations: Department of Molecular Medicine and Medical Biotechnology, University of Naples Federico II, 80131 Naples, Italy

Materials and Methods

Antibodies and reagents

Mouse anti-LAMP1 monoclonal antibody (555798) was purchased from BD Biosciences; mouse anti-β-actin monoclonal antibody (G043) from Abm; mouse anti-γ-tubulin antibody (T6557) from Sigma Aldrich; goat anti-mouse IgG polyclonal antibody conjugated to horseradish peroxidase (HRP) (sc-2031) from Santa Cruz Biotechnology; bovine serum albumin (BSA) (A7906), from Sigma Aldrich; SDS-PAGE reagents from Bio-Rad; fetal bovine serum (FBS) from GIBCO; Fibronectin (F2006) was purchased from Sigma Aldrich.

Cell cultures

Fibroblasts from MPS affected patients were kindly provided by the "Cell Line and DNA Biobank From Patients Affected by Genetic Diseases" (Istituto G. Gaslini, Genoa, Italy). Fibroblasts were cultured in Dulbecco's modified Eagle's medium (DMEM), supplemented with 10 % FBS, 2 mM L-glutamine, 100 Units/ml penicillin, and 100 μg/ml streptomycin, at 37 °C in a humidified 5% CO_2 atmosphere.

Measurement of radioactive glucosamine content into GAG chains

MPS fibroblasts were grown in normal medium supplemented with 7 μCi/ml ^{3}H-glucosamine (PerkinElmer) up to 80% confluence. The radioactive medium was removed and cells were incubated for 48 h in normal medium containing 2% FBS and 10^{-6} M of fibronectin. Cells were washed with PBS before harvesting, suspended in water, and lysed using freeze thaw cycles. An aliquot of cell lysate was taken out for the determination of protein concentration using the Lowry method. Lipids were extracted by addition of chloroform and methanol (chloroform-methanol-water 4:8:3, v/v/v). After 10 min of incubation at room temperature, extracts were recovered by centrifugation (10000 g for 10 min), washed with acetone, dried, and subjected to proteolysis overnight at 65 °C with 1 mg/ml papain in 100 mM sodium acetate buffer containing 5 mM EDTA and 5 mM cysteine (pH 5.5). The content of ^{3}H-glucosamine was measured by liquid scintillation counting, and normalized against protein concentration.

Western blotting

Protein extraction from cell lysates and immunoblot was performed as previously described. Briefly, fibroblasts were harvested in lysis buffer (50 mM Tris pH 7.5, 150 mM NaCl, 1 mM EDTA, 1 mM EGTA, 10 % glycerol, 1 % Triton-X-100, 1 mM β-glycerophosphate, 1 mM phenylmethylsulfonyl fluoride, protease inhibitor cocktail tablet, 1 mM sodium orthovanadate, 2.5 mM sodium pyrophosphate), incubated for 30 min on ice, and supernatants were collected and centrifuged for 10 min at 14,000 g. Protein concentration was estimated by Bradford assay, and 50

μg/lane of total proteins were separated on SDS gels and transferred to nitrocellulose membranes. Membranes were treated with a blocking buffer (25 mM Tris, pH 7.4, 200 mM NaCl, 0.5% Triton X-100) containing 5% non-fat powdered milk for 1 h at room temperature). Incubation with the primary antibody was carried out overnight at 4 °C. After serial washings, membranes were incubated with the HRP-conjugated secondary antibody for 1 h at room temperature. Following further washings of the membranes, chemiluminescence was generated by ECL system.

Statistical analysis

Data reported are expressed as the mean ± standard deviation (S.D.) of at least three separate experiments. Statistical significance was determined by Student's t-test. The value of $P < 0.05$ was considered to be statistically significant.

SUPPLEMENTARY FIGURE LEGENDS

Figure S1. A. Effect of fibronectin on ^{3}H-glucosamine content into GAGs of fibroblasts from MPS IIIB patients. Data reported are the means ± S.D. of three independent experiments performed in triplicate. *P<0.05. **B.** LAMP1 protein expression levels in untreated and NK1-treated MPS I and MPS IIIB fibroblasts as measured by Western blotting analysis. The upper blots were stripped and re-probed with anti-γ-tubulin or anti-β-actin antibodies to ensure equal loading of proteins in all lanes. The blots reported are representative of three independent experiments of equal design.

Figure S1

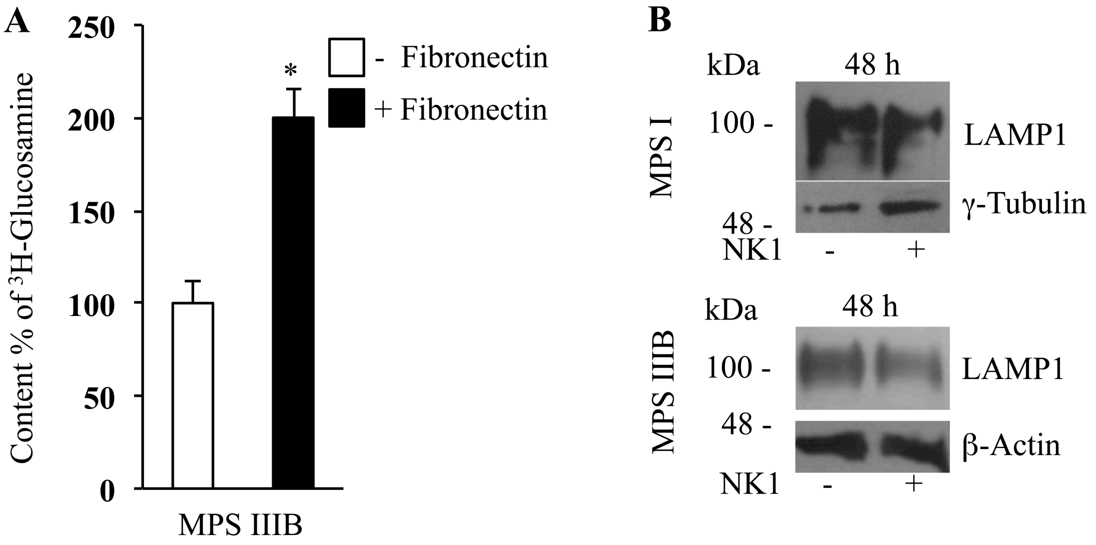

Supplement: Document S2. Article plus Supplemental Information [file mmc2.pdf]
